# Advances in pediatric video capsule endoscopy: current applications and future directions

**DOI:** 10.3389/fped.2025.1738998

**Published:** 2026-01-22

**Authors:** Isabel Rojas, Bradley A. Barth, Jeremy W. Stewart

**Affiliations:** Division of Pediatric Gastroenterology, Hepatology and Nutrition, University of Texas Southwestern Medical Center, Dallas, TX, United States

**Keywords:** artificial intelligence, magnetically controlled capsule, patency capsule, pediatric gastroenterology, video capsule endoscopy, wireless motility capsule

## Abstract

Video capsule endoscopy (VCE) has revolutionized the evaluation of small bowel pathology, offering a safe, non-invasive, radiation-free diagnostic modality with broad clinical utility. Patency capsule use has further improved safety by minimizing the risk of retention in patients with suspected strictures. Since its introduction, its applications have expanded from obscure gastrointestinal bleeding and Crohn's disease to celiac disease, polyposis syndromes, and small bowel tumors among other indications. Emerging artificial intelligence (AI) integration promises to enhance diagnostic accuracy, streamline image analysis, and reduce interobserver variability. Furthermore, advancements in capsule design, including magnetic-assisted navigation and extended battery life, enable precise control and complete small bowel evaluation, even in cases of delayed gastrointestinal motility. High-definition imaging further allows for the identification of subtle mucosal abnormalities, such as vascular lesions, inflammation, and erosions, that might otherwise go undetected. Beyond diagnosis, novel applications, such as motility capsule studies and wireless capsule drug delivery systems, are unlocking new possibilities for functional and therapeutic interventions. Future innovations combining diagnostic and interventional capabilities promise to reduce the need for invasive procedures, optimize outcomes, and significantly enhance the quality of life for pediatric patients.

## 1 Introduction

Video capsule endoscopy (VCE) has transformed the diagnostic landscape of small bowel evaluation since its initial introduction in 2000. Its non-invasive nature, lack of ionizing radiation, and ability to directly visualize the entire small intestine make it particularly well suited for use in pediatric populations, where minimizing procedural risk and patient discomfort is paramount (1, 2). Initially developed to investigate obscure gastrointestinal bleeding in adults, VCE has since found widespread applications in children, including the evaluation of suspected Crohn's disease, celiac disease, polyposis syndromes, and small bowel tumors (3).

Pediatric gastrointestinal disorders often present with nonspecific symptoms and require comprehensive evaluation of the small bowel; an area historically considered a diagnostic blind spot. Traditional modalities such as radiographic imaging, enteroscopy, and surgery carry limitations in sensitivity, invasiveness, and feasibility in children. VCE bridges this gap by enabling detailed mucosal assessment with high diagnostic yield, particularly in cases where other tests have been inconclusive (1, 4, 5).

In recent years, technological advances, including high-definition imaging, extended battery life, and novel delivery systems, have further expanded the scope and effectiveness of VCE in pediatric care (6, 7). The integration of artificial intelligence (AI) into image analysis platforms is poised to enhance lesion detection, reduce reading times, and minimize interobserver variability (8, 9). Simultaneously, safety innovations such as the use of patency capsules help mitigate the risk of capsule retention in children with suspected strictures, broadening the patient populations for whom VCE can be safely performed (10, 11).

As VCE continues to evolve, its role is shifting from purely diagnostic to potential therapeutic and functional applications. This review provides an update on the current clinical use of VCE in children, highlights key technological and safety advancements, and explores future directions that may redefine its place in pediatric gastroenterology.

## 2 Current clinical applications in children

**Figure 1:**
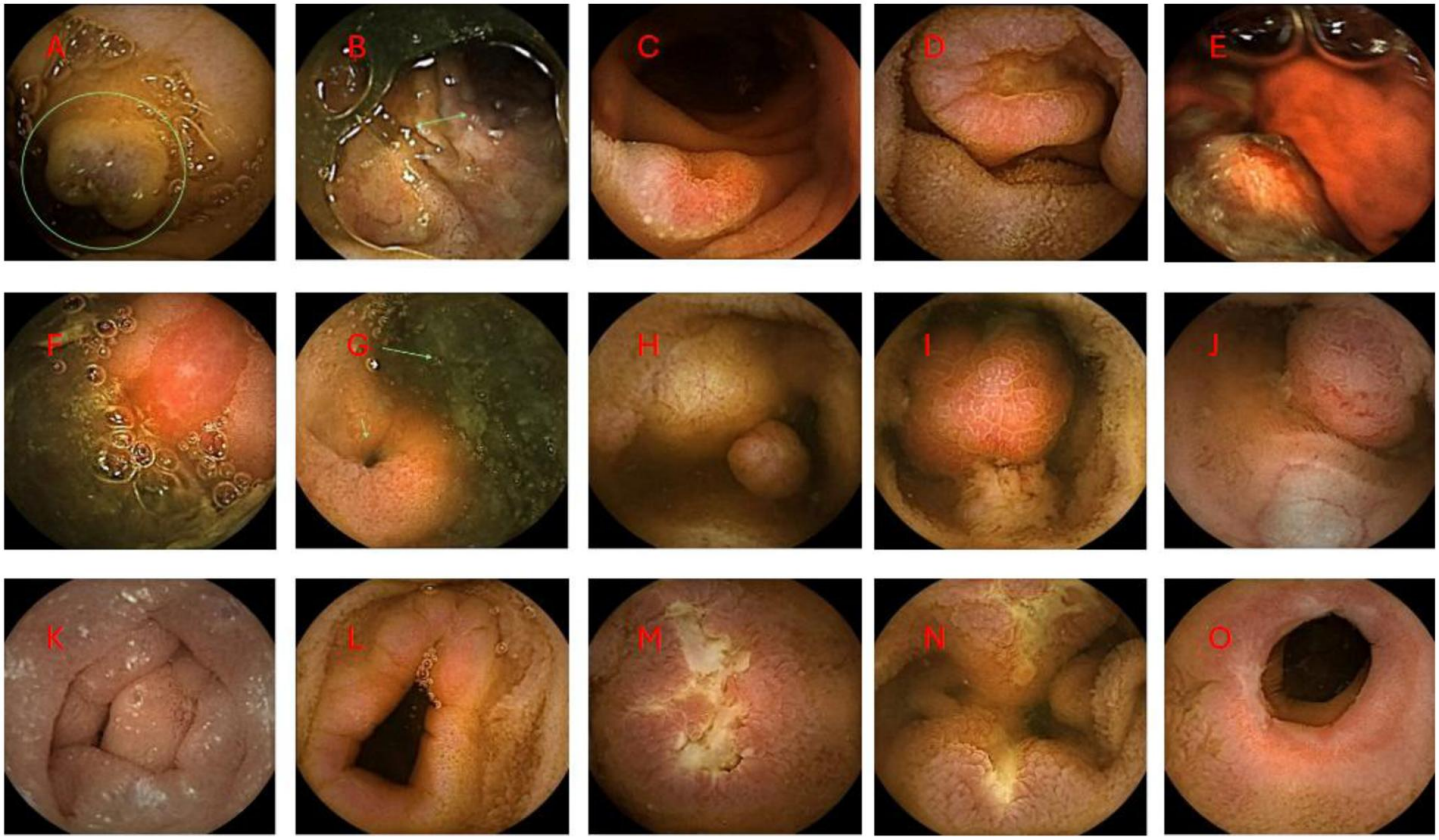
Representative capsule endoscopy findings in pediatric patients. **(A,B)** Vascular malformations: **(A)** bleb-like lesion; **(B)** serpiginous vascular malformation (arrow). **(C,D)** Jejunal ulcers in a heart transplant recipient, subsequently diagnosed with post-transplant lymphoproliferative disorder. **(E)** Ulcer with active bleeding. **(F)** Anastomotic ulcer in a patient with a history of small bowel resection. **(G)** Double-lumen sign (arrows) in a patient diagnosed with Meckel's diverticulum. **(H–J)** Small and large polyps in patients with Peutz-Jeghers syndrome. **(K)** White-tipped and engorged villi in a patient with protein-losing enteropathy. **(L)** Jejunal narrowing with associated inflammatory changes in a patient with eosinophilic enteritis. **(M,N)** Ulcers in patients with small bowel Crohn's disease. **(O)** Stricture in a patient with small bowel Crohn's disease.

Video capsule endoscopy (VCE) has become an indispensable diagnostic tool for evaluating small bowel pathology in children (Figure 1). Its ability to provide high-resolution mucosal visualization without the need for sedation or ionizing radiation makes it especially valuable in children, where conventional modalities are limited. Although pediatric data remains more limited than adult studies, evidence consistently supports its safety, feasibility, and diagnostic yield across a range of clinical indications (1, 12–14).

### 2.1 Obscure gastrointestinal bleeding (OGIB)

Obscure gastrointestinal bleeding remains the most frequent indication for VCE in children, particularly those under eight years of age. This modality is highly effective in identifying sources of occult or overt bleeding that go undetected following negative upper and lower endoscopies. Reported diagnostic yields vary widely from approximately 19%–95% in small series, with a multicenter pediatric study demonstrating a yield of around 53% (1, 2). Common findings include vascular malformations, ulcers, erosions, and occasionally Meckel's diverticulum (12, 13). In many cases, VCE findings directly inform subsequent management, including therapeutic intervention via device-assisted enteroscopy. Although adult data suggest that earlier timing of capsule administration, within 24–72 h of a bleeding episode, increased diagnostic yield, pediatric evidence supporting this interval is limited; thus, its application should be considered extrapolated from adult studies or center-specific practice (1).

### 2.2 Crohn's disease

Video capsule endoscopy (VCE) plays a pivotal role in the evaluation and management of suspected or established Crohn's disease. It enables direct visualization of early or isolated small bowel lesions that may be missed by conventional endoscopy or imaging. The North American Society for Pediatric Gastroenterology, Hepatology and Nutrition (NASPGHAN) recognizes VCE as a key modality for identifying small bowel involvement, assessing disease extent and recurrence, and guiding treatment decisions. Capsule endoscopy demonstrates good sensitivity compared with magnetic resonance enterography (MRE) and computed tomography enterography (CTE), with variable specificity (1). Prospective data support complementary roles for VCE and MRE; VCE offers higher specificity for mucosal disease and can influence management decisions, including monitoring for mucosal healing within treat-to-target paradigms (15, 16). Pediatric cohorts further document that VCE findings lead to therapeutic modifications in a substantial proportion of inflammatory bowel disease cases (14).

### 2.3 Celiac disease

While histology remains the diagnostic standard, VCE can identify characteristic features such as villous atrophy, scalloping, and mosaic pattern, and is particularly useful when esophagogastroduodenoscopy (EGD) is not feasible or when assessing disease extent or complications. Clinical studies report high performance for detecting villous atrophy (17). A contemporary meta-analysis further supports substantial detection of these features, with higher diagnostic yield in refractory cases, reinforcing VCE's role as an adjunctive tool (18).

### 2.4 Polyposis syndromes

Capsule endoscopy is employed for small bowel surveillance in pediatric hamartomatous polyposis syndromes, particularly Peutz-Jegher's syndrome, allowing assessment of small-bowel polyp burden and guiding the timing of device-assisted enteroscopy. The European Society for Paediatric Gastroenterology, Hepatology and Nutrition (ESPGHAN) recommends small bowel surveillance using VCE and/or MRE beginning no later than 8 years, with VCE demonstrating greater sensitivity than imaging modalities (19).

### 2.5 Small bowel tumors

Direct visualization of mucosal abnormalities, including masses, ulcerations, and polypoid lesions, is achievable with VCE, making it particularly useful for detecting lesions missed by radiography or cross-sectional imgaing and facilitating early diagnosis as well as guiding subsequent management, such as device-assisted enteroscopy or surgery (20). In pediatric cohorts, VCE has demonstrated high completion rates and diagnostic yield across a range of small bowel pathologies, including rare tumors, with abnormal findings reported in up to 59% of cases (12–14). NASPGHAN recommends VCE as a complementary tool when other modalities fail to identify a lesion (1).

### 2.6 Other emerging indications

Additional indications for VCE in children include protein-losing enteropathy, graft-vs.-host disease, and surveillance in post-operative or radiation-induced enteropathy (1, 21). With ongoing innovation and increasing familiarity, the range of indications is expected to expand further.

## 3 Capsule retention and the role of patency capsule

### 3.1 Capsule retention

Capsule retention in pediatric VCE occurs in approximately 1%–2% of procedures overall, with higher rates reported among children with known or suspected Crohn's disease, small bowel strictures, or post-surgical anatomy. Retention is most often asymptomatic but may present with abdominal pain, nausea, or signs of bowel obstruction. The use of a patency capsule offers a safe and effective method to assess small bowel patency in at-risk pediatric populations, such as those with Crohn's disease, previous intestinal surgery, or suspected stricturing disease. Traditional screening approaches, based on clinical history or cross-sectional imaging, demonstrate limited sensitivity, whereas the patency capsule provides superior specificity and a lower false-negative rate.

Large pediatric series and meta-analyses have reported overall capsule retention rates between 1.4% and 2.3%, with higher frequencies observed in Crohn's disease (up to 2.2%–2.6%) and in patients with strictures. Retention most commonly results from inflammatory or post-surgical narrowing, and rates are determined primarily by indication rather than age (1, 4, 11, 22). Although often clinically silent, capsule retention can cause obstruction or, rarely, perforation; symptoms may include abdominal pain, nausea, or vomiting, and surgical intervention is occasionally required (1).

### 3.2 Risk assessment

Conventional risk stratification methods, including review of clinical history, MR or CT enterography, and fluoroscopic studies, can help identify children at risk for retention, but these techniques have limited sensitivity for detecting functional patency. Cross-sectional imaging has a pooled sensitivity of 54% and a specificity of 88%, for predicting retention, while the patency capsule demonstrates higher specificity (94%) and lower false-negative rates of 2.7% (23).

### 3.3 Patency capsule mechanism and utility

The patency capsule (e.g., Agile Patency Capsule) is a dissolvable, radio-opaque capsule that mimics the size and shape of a VCE device. It contains an internal time plug that causes the capsule to disintegrate after a predetermined time, typically 30–40 h, breaking down into small fragments, allowing it to pass safely through the intestine, thereby minimizing the risk of obstruction. It contains a radiofrequency identification (RFID) tag or similar marker that permits noninvasive detection of the capsule's location using an external scanner or imaging (24).

It is recommended for children at increased risk of small bowel strictures, including those with Crohn's disease, prior bowel surgery, or post-inflammatory or radiation-induced narrowing. It is safe and feasible in older children and adolescents, with multicenter studies demonstrating high successful passage rates and minimal adverse events (4, 24–26). Overall, the patency capsule provides a more reliable assessment of small bowel patency compared with imaging alone and effectively predicts safe passage of the diagnostic capsule (23, 26).

## 4 Technological advancements

### 4.1 High-definition imaging

**Figure 2:**
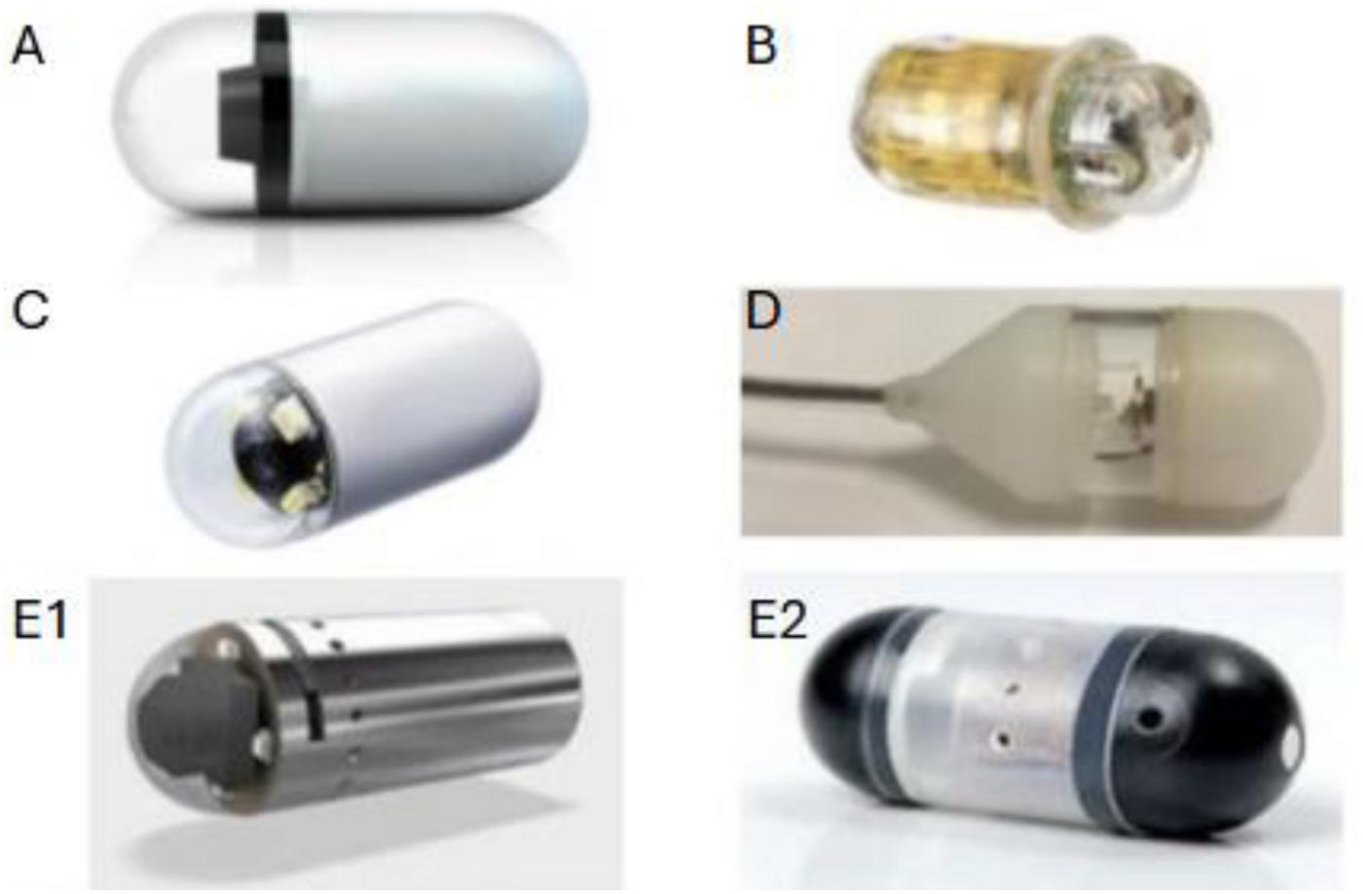
Representative examples of capsule endoscopy devices and emerging technologies. **(A)** Standard third-generation VCE: conventional diagnostic capsule used for small bowel imaging, equipped with dual cameras and LED illumination. **(B)** Motility capsule: measures intraluminal pH, pressure, and temperature to assess regional and whole-gut transit times. **(C)** Magnetically controlled capsule: contains embedded magnetic material allowing external guidance and targeted visualization of the upper GI tract. **(D)** Tethered capsule: attached to a thin flexible tether, provides real-time imaging while controlled movement and retrieval. E1 and E2. Prototype therapeutic or hybrid capsules: examples include site-specific drug delivery systems and interventional capsules with capabilities such as biopsy or hemostasis.

Third-generation capsules, such as PillCam SB3 (Figure 2), incorporate high-resolution sensors and adaptive frame rate technology, substantially improving the visualization of subtle mucosal abnormalities, including aphthous ulcers, erosions, or vascular lesions. Enhanced image clarity facilitates earlier and more accurate detection of small bowel disease and increases interobserver agreement in pediatric interpretation. Integration of advanced optics and software-based image enhancement has further optimized contrast and mucosal detail, improving diagnostic confidence in children with suspected inflammatory or vascular pathology (1, 6, 7) (Table 1).

**Table 1:** Key technological advancements in VCE relevant to pediatric use.

Advancement	Description/Example	Pediatric Impact	Current Limitations
High-definition imaging	Enhanced sensor resolution and optical systems (e.g., PillCam HD)	Improved mucosal visualization and lesion detection	Increased data size and reading time
Extended battery life	Next-gen capsules with >12–14 h runtime	Enables complete small-bowel visualization in slower transit	Slightly larger capsule dimensions
Real-time viewing	Bluetooth or wireless transmission for live monitoring	Allows early identification of retention or incomplete exams	Limited to select models
Magnetically guided navigation	External magnetic control for directed capsule movement	Potentially reduces incomplete exams; safe in children	Requires specialized equipment
Pediatric capsule sizes	Smaller capsules and oral delivery devices	Increases feasibility in younger children	Limited commercial availability
AI-assisted software	Automated bleeding/lesion detection and image triage	Reduces reading time, increases accuracy	Mostly validated in adults

### 4.2 Extended battery life

Extended battery life in newer capsule models (Pillcam SB2-ex, SB3) is associated with higher rates of complete small bowel transit and fewer incomplete studies, which is especially important in children with slower transit times or anatomical variations (7). Large pediatric cohorts report completion rates exceeding 95%, supporting the clinical impact of longer battery duration (12–14).

### 4.3 Magnetically guided capsule systems

Magnetically controlled capsule endoscopy (MCE) represents a significant innovation, enabling active navigation and targeted visualization of the upper gastrointestinal tract, overcoming the limitations of passive capsule transit, and without the need for sedation. While its application in pediatrics remains limited, feasibility studies demonstrate safe use and excellent visualization, facilitating gastric emptying and transpyloric passage, including children as young as 6 years (27, 28). The ability to retrieve or reposition capsules in real-time enhances procedural safety, a crucial advantage in pediatrics (Figure 2).

### 4.4 Real-time viewing capabilities

Real-time viewing allows immediate assessment of capsule location and mucosal findings, improving procedural efficiency, and enabling prompt intervention if needed. This feature is highlighted in the NASPGHAN report and recent pediatric studies as beneficial for workflow and safety (1).

### 4.5 Pediatric-specific capsule sizes and delivery systems

The development of smaller capsule sizes and improved delivery accessories has expanded the use of VCE in children as young as 8 months of age and as light as 7.9 kg (1, 29, 30). Dedicated pediatric delivery devices, such as endoscopic capsule deployment systems (e.g., AdvanCE), enable safe administration in patients unable to swallow the capsule, including those under six years of age (2, 30–32). These tailored designs have increased procedural success and safety, broadening the applicability of capsule accuracy across the pediatric age spectrum.

## 5 Artificial intelligence in video capsule endoscopy

### 5.1 Overview of artificial intelligence integration

Artificial intelligence (AI) has rapidly emerged as one of the most transformative developments in VCE. With each study producing tens of thousands of images, manual review is time-sensitive and subject to human fatigue and variability. AI-driven algorithms, particularly those based on convolutional neural networks (CNNs), are being developed to assist in automatic detection, classification, and localization of small bowel lesions. These systems are designed to augment, rather than replace, physician interpretation, thereby improving efficiency, diagnostic consistency, and consistency across readers. Multiple multicenter studies and systematic reviews have confirmed that deep learning models, especially CNN-based architectures, can automatically detect and classify small bowel lesions and localize findings within the gastrointestinal tract, providing strong evidence for their use as reliable physician-assistive tools (8, 33).

### 5.2 Applications: lesion detection, bleeding identification, image prioritization

AI integration in capsule endoscopy primarily focuses on automatic lesion recognition, including ulcers, erosions, angioectasias, and polyps. Algorithms trained on large annotated datasets can identify pathological features with sensitivities and specificities comparable to expert reviewers. Deep-learning systems have demonstrated excellent accuracy in detecting small bowel bleeding and vascular lesions, automatically flagging relevant frames for clinician review and prioritizing high-yield segments of the video. Some systems also provide lesion localization and quantification, facilitating longitudinal disease monitoring in conditions such as Crohn's disease. Meta-analyses and large validation studies consistently report that CNN-based algorithms achieve sensitivities and specificities exceeding 95% for the detection of ulcers, bleeding, and polyps, often matching or surpassing expert human performance. These AI models reliably identify a wide range of lesions relevant to both pediatric and adult populations, reinforcing their role as powerful assistive tools in capsule interpretation (8, 34).

### 5.3 Benefits: reduced reading time, improve accuracy, minimized interobserver variability

AI-assisted reading has demonstrated substantial improvements in efficiency and diagnostic consistency. Multiple studies report dramatic reductions in interpretation time, from 60 to 96 min to as little as 5–15 min, without compromising, and in some cases improving, diagnostic accuracy (8, 35, 36). By automatically highlighting clinically relevant frames, AI minimizes the risk of missed lesions and enhances detection consistency across readers with varying experience levels. This standardization of review reduces interobserver variability and increases reproducibility, benefits that are particularly valuable in pediatric centers where capsule interpretation may be performed by clinicians with differing levels of expertise (9).

### 5.4 Current tools available and pediatric data

**Figure 3:**
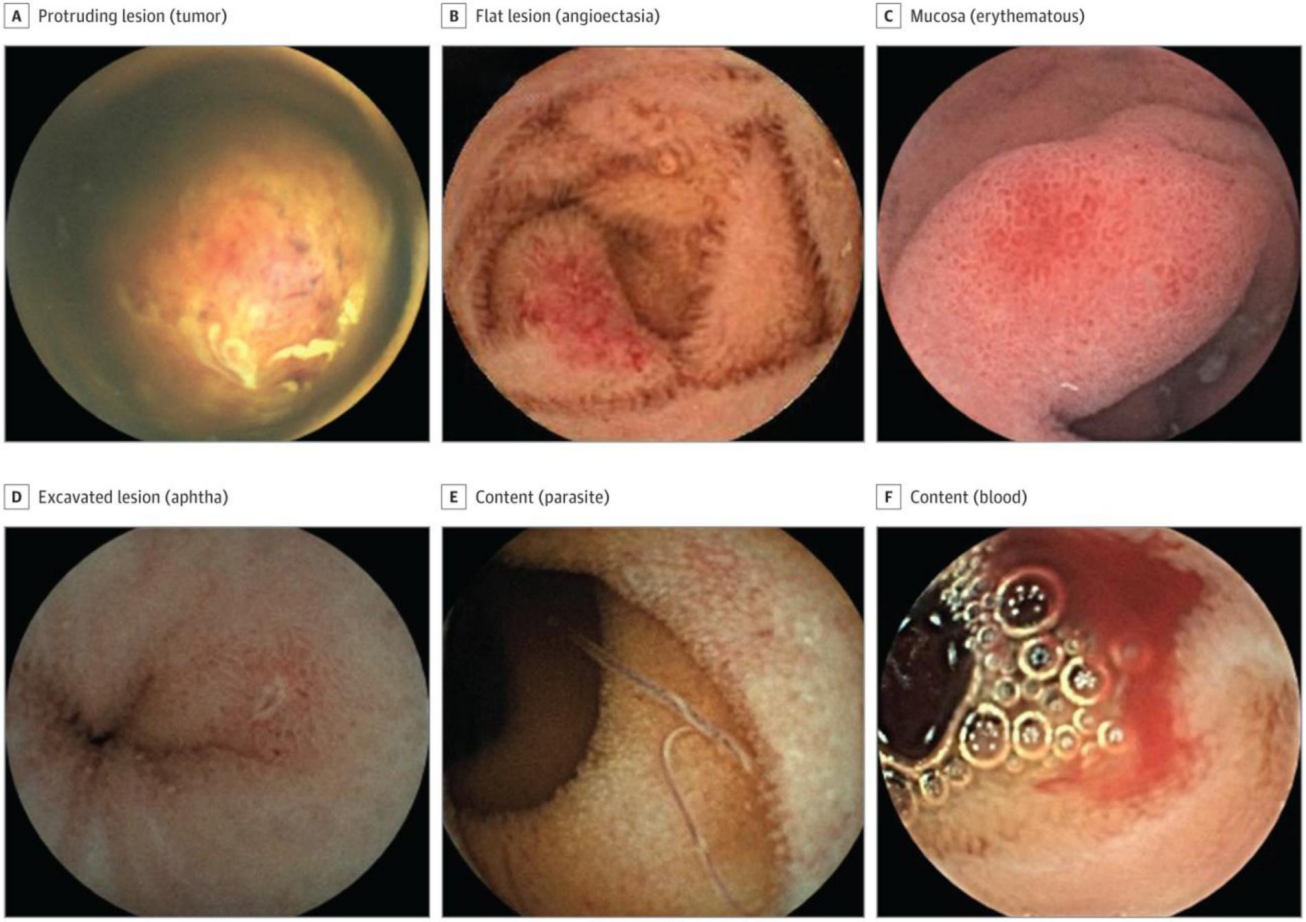
Findings detected by SmartScan-assisted Reading [Ref. (36), CC-BY license]. **(A)** Protruding lesion (tumor). **(B)** Flat lesion (angioectasia). **(C)** Mucosa (erythematous). **(D)** Excavated lesion (aphtha). **(E)** Content (parasite). **(F)** Content (blood).

Commercial AI-enabled capsule platforms, including Omni Mode (Medtronic) and SmartScan (IntroMedic), are now integrated into clinical software, offering automated bleeding detection, lesion identification, and image triage capabilities (Figure 3) (36, 37). Most validation studies have been conducted in adult cohorts, where these systems have shown strong diagnostic performance and substantial reductions in review time. Although pediatric-specific data remain limited, extrapolation of results to children is reasonable given the similarity in lesion morphology and imaging characteristics. Preliminary pediatric experience suggests that AI-assisted analysis can enhance workflow efficiency and diagnostic confidence, but larger, dedicated pediatric studies are needed to validate performance across younger age groups and rare disease contexts (37).

## 6 Novel and future applications

### 6.1 Motility capsule studies

Wireless motility capsules (Figure 2), such as the *SmartPill*, measure intraluminal pH, pressure, and temperature to evaluate regional and whole gut transit times, offering valuable data for the assessment of gastrointestinal motility disorders without radiation exposure. Their use in pediatrics remains limited due to capsule size, regulatory restrictions, and the absence of validated pediatric normative data. Studies in adolescents and older children (ages 8–17) have demonstrated feasibility and safety, but practical limitations, including difficulty swallowing the capsule and variable gastric emptying, restrict use in younger patients. Ongoing efforts to miniaturize capsule devices and establish age-appropriate reference ages are underway, with the potential to integrate motility assessment into routine capsule-based diagnostics in the near future (38–41) (Table 2).

**Table 2:** Emerging capsule technologies and potential applications in pediatrics.

Capsule Type/Technology	Primary Function	Current Development Stage	Pediatric Relevance
Wireless motility capsule (e.g., SmartPill)	Measures pH, pressure, and temperature for whole-gut transit	Clinical use (adolescents); limited in younger children	Noninvasive motility assessment without radiation
Drug delivery capsule	Site-specific release via pH/magnetic trigger	Preclinical/early translational	Potential for targeted therapy in IBD
Hybrid diagnostic-interventional capsule	Combines imaging with biopsy, cautery, or hemostasis	Experimental prototypes	Could minimize need for anesthesia and endoscopy
Magnetically controlled capsule	Real-time navigation and retrieval	Clinical trials (upper GI)	Improves safety, allows active control in children
Tethered capsule	Real-time imaging, immediate retrieval	Feasibility studies	Safe, avoids sedation; may replace some endoscopic exams

### 6.2 Wireless capsule drug delivery systems

Beyond diagnostics, capsule technology is advancing toward targeted therapy. Wireless drug delivery capsules are being developed to release medication at specific gastrointestinal sites using pH, enzymatic, or magnetic triggers. Experimental models have demonstrated the feasibility of site-specific delivery for anti-inflammatory agents, antibiotics, and biologics, offering distinct advantages for diseases with localized pathology such as Crohn's disease. For pediatric patients, these systems could minimize systemic exposure and improve adherence by combining diagnosis and treatment within a single, noninvasive platform. Although still in preclinical or early transitional stages, continued progress in miniaturization and biocompatible materials to facilitate future pediatric applications (42–44).

### 6.3 Hybrid diagnostic-interventional capsules

Next-generation “active” capsule systems aim to integrate diagnostic imaging with interventional functions such as biopsy, electrocautery, and hemostatic delivery (Figure 2). Prototype devices have demonstrated proof of concept for tissue sampling, bleeding control, and localized therapy without the need for conventional endoscopy. These multifunctional capsules represent a potential paradigm shift in minimally invasive gastroenterology, particularly valuable in pediatrics, where reducing anesthesia exposure and procedural invasiveness is a major priority. While translational to clinical use remains forthcoming, rapid progress in micro-robotics, wireless actuation, and energy control continues to expand the feasibility of these platforms (42–44).

### 6.4 Tethered capsules and magnetically controlled navigation

Tethered capsule endoscopes are under active investigation, offering real-time and controlled movement through the gastrointestinal tract via external magnetic or manual guidance (Figure 2). These devices typically include a thin tether for retrieval or manipulation and can be combined with magnetic steering for enhanced precision. Cable-transmission magnetically controlled capsule endoscopy (CT-MCCR) systems have shown high diagnostic yield and safety for upper GI evaluation, with performance comparable to conventional gastroscopy and improved patient tolerance (28, 45, 46).

## 7 Discussion

Video capsule endoscopy has become an essential, minimally invasive tool for evaluating small bowel pathology in children, particularly for obscure gastrointestinal bleeding, inflammatory bowel disease, celiac disease, and hereditary polyposis syndromes. Advances in capsule design, including smaller size, extended battery life, and patency testing, have improved safety and broadened applicability. However, most technical validation originates from adult populations, and pediatric-specific data remain limited. Differences in anatomy, motility, and disease presentation highlight the need for dedicated pediatric studies to refine protocols and establish normative standards.

Artificial intelligence (AI)-assisted reading represents one of the most promising developments in capsule endoscopy. Early data show that AI can reduce reading time, enhance diagnostic accuracy, and minimize interobserver variability. Commercial systems with integrated AI triage and lesion detection are now available, but pediatric validation is scarce. The creation of large, annotated pediatric image databases through multicenter collaboration will be essential to ensure algorithm reliability across different ages and disease phenotypes.

Emerging technologies are transforming VCE from a passive diagnostic tool into an active, multifunctional platform. Motility capsules enable radiation-free assessment of gastrointestinal transit, while wireless drug delivery and hybrid diagnostic-interventional capsules may one day permit targeted therapy and tissue sampling without conventional endoscopy. Magnetically controlled and tethered capsule systems, especially when coupled with AI-based navigation, allow real-time steering, retrieval, and optimized visualization, reducing the need for sedation and improving procedural safety.

In summary, capsule endoscopy continues to evolve rapidly within pediatric gastroenterology. The convergence of miniaturization, smart navigation, and AI-driven interpretation promises to expand diagnostic reach and therapeutic potential while maintaining a noninvasive approach. Ongoing efforts to validate these technologies in children and ensure equitable access will be crucial to realizing the full clinical impact of next-generation capsule endoscopy.

## References

[1] Friedlander JA Liu QY Sahn B Kooros K Walsh CM Kramer RE NASPGHAN capsule endoscopy clinical report. J Pediatr Gastroenterol Nutr. (2017) 64:485–94. 10.1097/MPG.0000000000001413 27642781

[2] Fritscher-Ravens A Scherbakov P Bufler P Torroni F Ruuska T Nuutinen H The feasibility of wireless capsule endoscopy in detecting small intestinal pathology in children under the age of 8 years: a multicentre European study. Gut. (2009) 58(11):1467–72. 10.1136/gut.2009.177774 19625281

[3] Wyllie R Hyams JS Kay M. Pediatric Gastrointestinal and Liver Disease Book. 7th edn. Philadelphia, PA: Elsevier (2025).

[4] Oliva S Cohen SA Di Nardo G Gualdi G Cucchiara S Casciani E. Capsule endoscopy in pediatrics: a 10-years journey. World J Gastroenterol. (2014) 20(44):16603–8. 10.3748/wjg.v20.i44.16603 25469028 PMC4248203

[5] Zevit N Shamir R. Wireless capsule endoscopy of the small intestine in children. J Pediatr Gastroenterol Nutr. (2015) 60(6):696–701. 10.1097/MPG.0000000000000782 (Erratum in: J Pediatr Gastroenterol Nutr. 2016;62(3):514.). 25782661

[6] Hosoe N Takabayashi K Ogata H Kanai T. Capsule endoscopy for small-intestinal disorders: current status. Dig Endosc. (2019) 31(5):498–507. 10.1111/den.13346 30656743

[7] Monteiro S de Castro FD Carvalho PB Moreira MJ Rosa B Cotter J. Pillcam® SB3 capsule: does the increased frame rate eliminate the risk of missing lesions? World J Gastroenterol. (2016) 22(10):3066–8. 10.3748/wjg.v22.i10.3066 26973404 PMC4779931

[8] Ding Z Shi H Zhang H Meng L Fan M Han C Gastroenterologist-level identification of small-bowel diseases and normal variants by capsule endoscopy using a deep-learning model. Gastroenterology. (2019) 157(4):1044–54.e5. 10.1053/j.gastro.2019.06.025 31251929

[9] Park J Hwang Y Nam JH Oh DJ Kim KB Song HJ Artificial intelligence that determines the clinical significance of capsule endoscopy images can increase the efficiency of Reading. PLoS One. (2020) 15(10):e0241474. 10.1371/journal.pone.0241474 33119718 PMC7595411

[10] Rosa B Dray X Koulaouzidis A. Retention of small bowel capsule endoscopy. Curr Opin Gastroenterol. (2023) 39(3):227–33. 10.1097/MOG.0000000000000921 37144540

[11] Pasha SF Pennazio M Rondonotti E Wolf D Buras MR Albert JG Capsule retention in Crohn’s disease: a meta-analysis. Inflamm Bowel Dis. (2020) 26(1):33–42. 10.1093/ibd/izz083 31050736

[12] Wu J Huang Z Wang Y Tang Z Lai L Xue A Clinical features of capsule endoscopy in 825 children: a single-center, retrospective cohort study. Medicine (Baltimore). (2020) 99(43):e22864. 10.1097/MD.0000000000022864 33120825 PMC7581167

[13] Li L Zhan X Li J Li S Chen Y Yang L Clinical assessment of small bowel capsule endoscopy in pediatric patients. Front Med (Lausanne). (2024) 11:1455894. 10.3389/fmed.2024.1455894 39478821 PMC11523533

[14] Liao YJ Lin WT Liao SC Lin SJ Huang YC Wu MC Clinical application and feasibility of capsule endoscopy in children at a medical center in central Taiwan. J Formos Med Assoc. (2025) 124(6):569–73. 10.1016/j.jfma.2024.06.012 38880710

[15] Hijaz NM Attard TM Colombo JM Mardis NJ Friesen CA. Comparison of the use of wireless capsule endoscopy with magnetic resonance enterography in children with inflammatory bowel disease. World J Gastroenterol. (2019) 25(28):3808–22. 10.3748/wjg.v25.i28.3808 31391775 PMC6676548

[16] Le Berre C Trang-Poisson C Bourreille A. Small bowel capsule endoscopy and treat-to-target in Crohn’s disease: a systematic review. World J Gastroenterol. (2019) 25(31):4534–54. 10.3748/wjg.v25.i31.4534 31496630 PMC6710184

[17] Rondonotti E Spada C Cave D Pennazio M Riccioni ME De Vitis I Video capsule enteroscopy in the diagnosis of celiac disease: a multicenter study. Am J Gastroenterol. (2007) 102(8):1624–31. 10.1111/j.1572-0241.2007.01238.x 17459022

[18] Shapiro M Niv Y. Diagnostic yield of video capsule endoscopy (VCE) in celiac disease (CD): a systematic review and meta-analysis. J Clin Gastroenterol. (2025) 59(7):598–606. 10.1097/MCG.0000000000002204 40434820

[19] Latchford A Cohen S Auth M Scaillon M Viala J Daniels R Management of Peutz-Jeghers syndrome in children and adolescents. J Pediatr Gastroenterol Nutr. (2019) 68:442–52. 10.1097/MPG.0000000000002248 30585892

[20] Fantasia S Cortegoso Valdivia P Kayali S Koulaouzidis G Pennazio M Koulaouzidis A. The role of capsule endoscopy in the diagnosis and management of small bowel tumors: a narrative review. Cancers (Basel). (2024) 16(2):262. 10.3390/cancers16020262 38254753 PMC10813471

[21] Varkey J Jonsson V Hessman E De Lange T Hedenström P Oltean M. Diagnostic yield for video capsule endoscopy in gastrointestinal graft- versus -host disease: a systematic review and metaanalysis. Scand J Gastroenterol. (2023) 58(8):945–52. 10.1080/00365521.2023.2175621 36740843

[22] Rezapour M Amadi C Gerson LB. Retention associated with video capsule endoscopy: systematic review and meta-analysis. Gastrointest Endosc. (2017) 85(6):1157–68.e2. 10.1016/j.gie.2016.12.024 28069475

[23] Kim YE Kim PH Yoon HM Lee JS Jung AY Cho YA Patency capsule and cross-sectional imaging for predicting capsule endoscopy retention: a systematic review and meta-analysis. Dig Dis Sci. (2025) 70(2):761–73. 10.1007/s10620-024-08835-6 39806086

[24] Herrerias JM Leighton JA Costamagna G Infantolino A Eliakim R Fischer D Agile patency system eliminates risk of capsule retention in patients with known intestinal strictures who undergo capsule endoscopy. Gastrointest Endosc. (2008) 67(6):902–9. 10.1016/j.gie.2007.10.063 18355824

[25] Odah T Karime C Hashash JG Kinnucan JA Picco MF Farraye FA. The utility of patency capsule in patients with Crohn’s disease. J Clin Gastroenterol. (2025) 59(6):562–8. 10.1097/MCG.0000000000002048 39729973

[26] Nakamura M Watanabe K Ohmiya N Hirai F Omori T Tokuhara D Tag-less patency capsule for suspected small bowel stenosis: nationwide multicenter prospective study in Japan. Dig Endosc. (2021) 33(1):151–61. 10.1111/den.13673 32215959

[27] Cheng W Lin K Wang L Wang X Feng Y Gu Z Clinical features of magnetically controlled capsule endoscopy in children: a large, retrospective cohort study. J Pediatr Gastroenterol Nutr. (2025) 80(4):733–41. 10.1002/jpn3.12472 39916493

[28] Di Nardo G Micheli F Cozzi DA Mercantini P Parisi P Baccini F Magnetic-assisted capsule endoscopy in children with Crohn disease: feasibility and impact on gastric transit time. J Pediatr Gastroenterol Nutr. (2023) 76(5):646–51. 10.1097/MPG.0000000000003733 36763990

[29] Nuutinen H Kolho KL Salminen P Rintala R Koskenpato J Koivusalo A Capsule endoscopy in pediatric patients: technique and results in our first 100 consecutive children. Scand J Gastroenterol. (2011) 46(9):1138–43. 10.3109/00365521.2011.584900 21615227

[30] Oikawa-Kawamoto M Sogo T Yamaguchi T Tsunoda T Kondo T Komatsu H Safety and utility of capsule endoscopy for infants and young children. World J Gastroenterol. (2013) 19(45):8342–8. 10.3748/wjg.v19.i45.8342 24363526 PMC3857458

[31] Iwama I Shimizu H Nambu R Okuhira T Kakuta F Tachibana N Efficacy and safety of a capsule endoscope delivery device in children. Eur J Gastroenterol Hepatol. (2019) 31(12):1502–7. 10.1097/MEG.0000000000001513 31464784

[32] Uko V Atay O Mahajan L Kay M Hupertz V Wyllie R. Endoscopic deployment of the wireless capsule using a capsule delivery device in pediatric patients: a case series. Endoscopy. (2009) 41(4):380–2. 10.1055/s-0029-1214491 19340746

[33] Aoki T Yamada A Kato Y Saito H Tsuboi A Nakada A Automatic detection of various abnormalities in capsule endoscopy videos by a deep learning-based system: a multicenter study. Gastrointest Endosc. (2021) 93(1):165–73.e1. 10.1016/j.gie.2020.04.080 32417297

[34] Qin K Li J Fang Y Xu Y Wu J Zhang H Convolution neural network for the diagnosis of wireless capsule endoscopy: a systematic review and meta-analysis. Surg Endosc. (2022) 36(1):16–31. 10.1007/s00464-021-08689-3 34426876 PMC8741689

[35] Andrade P Mascarenhas M Mendes F Rosa B Cardoso P Afonso J AI-assisted capsule endoscopy for detection of ulcers and erosions in crohn’s disease: a multicenter validation study. Clin Gastroenterol Hepatol. (2025):S1542-3565(25)00861-4(25)00861-4. 10.1016/j.cgh.2025.09.036 41076040

[36] Xie X Xiao Y-F Zhao X-Y Li J-J Yang Q-Q Peng X Development and validation of an artificial intelligence model for small bowel capsule endoscopy video review. JAMA Netw Open. (2022) 5(7):e2221992. 10.1001/jamanetworkopen.2022.21992 35834249 PMC9284338

[37] Giordano A Romero-Mascarell C González-Suárez B Guarner-Argente C. Integration of artificial intelligence-enhanced capsule endoscopy in clinical practice: a review of market-available tools for clinical practice. Dig Dis Sci. (2025) 70(9):2966–76. 10.1007/s10620-025-09099-4 40490597 PMC12411590

[38] Rodriguez L Heinz N Colliard K Amicangelo M Nurko S. Diagnostic and clinical utility of the wireless motility capsule in children: a study in patients with functional gastrointestinal disorders. Neurogastroenterol Motil. (2021) 33(4):e14032. 10.1111/nmo.14032 33184926

[39] Fritz T Hünseler C Broekaert I. Assessment of whole gut motility in adolescents using the wireless motility capsule test. Eur J Pediatr. (2022) 181(3):1197–204. 10.1007/s00431-021-04295-6 34786599 PMC8897340

[40] Green AD Belkind-Gerson J Surjanhata BC Mousa H Kuo B Di Lorenzo C. Wireless motility capsule test in children with upper gastrointestinal symptoms. J Pediatr. (2013) 162(6):1181–7. 10.1016/j.jpeds.2012.11.040 23290514

[41] Rybak A Martinelli M Thapar N Van Wijk MP Vandenplas Y Salvatore S Colonic function investigations in children: review by the ESPGHAN motility working group. J Pediatr Gastroenterol Nutr. (2022) 74(5):681–92. 10.1097/MPG.0000000000003429 35262513

[42] Rehan M Al-Bahadly I Thomas DG Young W Cheng LK Avci E. Smart capsules for sensing and sampling the gut: status, challenges and prospects. Gut. (2023) 73(1):186–202. 10.1136/gutjnl-2023-329614 37734912 PMC10715516

[43] Cummins G. Smart pills for gastrointestinal diagnostics and therapy. Adv Drug Deliv Rev. (2021) 177:113931. 10.1016/j.addr.2021.113931 34416311

[44] Hoffmann SV O’Shea JP Galvin P Jannin V Griffin BT. State-of-the-art and future perspectives in ingestible remotely controlled smart capsules for drug delivery: a GENEGUT review. Eur J Pharm Sci. (2024) 203:106911. 10.1016/j.ejps.2024.106911 39293502

[45] Tian Y Du S Liu H Yu H Bai R Su H Prospective, multicenter, self-controlled clinical trial on the effectiveness and safety of a cable-transmission magnetically controlled capsule endoscopy system for the examination of upper GI diseases (with video). Gastrointest Endosc. (2025) 101(4):804–17.e1. 10.1016/j.gie.2024.07.028 39111392

[46] Jiang B Qian YY Wang YC Pan J Jiang X Zhu JH A novel capsule endoscopy for upper and mid-GI tract: the UMGI capsule. BMC Gastroenterol. (2023) 23(1):76. 10.1186/s12876-023-02696-5 36927462 PMC10019395

